# Subcutaneous pedicled propeller flap for reconstructing the large eyelid defect due to excision of malignancies or trauma

**DOI:** 10.1038/s41598-022-09100-4

**Published:** 2022-03-22

**Authors:** Xiao-Ni Wang, Yu-Xi Tang, Tao Guo, Hai-Dong Hu, Qiang Ma, Bao-Fu Yu, Xiang-Dong Zhao

**Affiliations:** 1 Department of Burn Plastic and Aesthetic Surgery, General Hospital of Ningxia Medical University, No. 804, Shengli South Street, Xingqing District, Yinchuan, 750001 China; 2 Department of Plastic and Reconstructive Surgery, Shanghai Ninth People’s Hospital, Shanghai Jiaotong University School of Medicine, Shanghai, 200011 China

**Keywords:** Surgical oncology, Skin diseases

## Abstract

Large eyelid defect after excision of malignancies or trauma is difficult to reconstruct due to special structure and function of the eyelid. In this study, we aimed to present the outcomes of subcutaneous pedicled propeller flap for reconstructing the large eyelid defect after excision of malignancies or trauma. A retrospective review of patients diagnosed with eyelid defect due to excision of malignancies or trauma, and undergoing subcutaneous pedicled propeller flap for reconstructing the large eyelid defect, was conducted at our hospital. The clinical data were collected and analyzed. A total of 15 patients were included in the cases series. Nine patients were diagnosed with basal cell carcinoma, 3 patients with epidermoid carcinoma, and 3 patients with trauma. All the defects were successfully covered with this designed flap. There was no flap necrosis in all the cases. No functional problems were observed in all of the cases. At long-term postoperative follow-up, the average score of patients’ satisfaction was good. This subcutaneous pedicled propeller flap is a feasible alternative technique for reconstructing the large eyelid defect after excision of malignancies or trauma. This flap option could avoid the use of free flaps for large defect.

## Introduction

Tumor resections, trauma and congenital defects are main factors resulting in eyelid defect^1,2^. Periocular cutaneous malignancies commonly include squamous cell carcinoma and basal cell carcinoma, and affect the eyelids and surrounding tissues^3^. The standard treatments typically include surgical excision, which usually breaks the normal anatomy and affects the function of eyelid^4–6^. Trauma of eyelid is also not rare in our clinical work. Depending on the size of defect, surgical reconstruction of the large eyelid defect to restore normal anatomy and function is necessary and challenging for surgeons. The surgical methods of reconstruction may have great effects on the clinical outcomes.

Protecting the ocular surface without affecting visual axis is the normal function of the eyelid. When reconstructing the defect of periocular region, ocular surface protection should be considered preferentially. Associated eyelid complications mainly include cicatricial ectropion, cicatricial entropion, eyelid retraction, notching, and so on^7,8^. When any of the complications happens, it would be very difficult to be corrected. Therefore, the best way is to avoid them when we perform the surgery at the first stage. Reconstructing of the defect must be meticulous. Avoiding vertical tension, addressing laxity and respecting aesthetic units should all be taken into considerations, when the surgery is planned and performed.

Auricular cartilage and palatal mucosa are common graft materials for reconstructing osterior lamella defect^9,10^. Besides, different kinds of local flaps, like cheek advancement flaps, transposition flaps, and VY flap procedures, have been reported to repair the eyelid defect^11–13^. However, there still exist many problems with the reconstructed eyelid. Ectropion, eyelid contracture, notching, and eyelid bulging are not rare after the reconstruction surgery^14,15^. There is not any ideal reconstruction method for all kinds of eyelid defect^16^. Besides, patient expectations have been increased nowadays. Therefore, it is necessary to explore more appropriate or alternative surgical techniques for reconstructing large eyelid defect.

In this study, we designed a subcutaneous pedicled propeller flap for reconstructing the large eyelid defect, which resulted from the excision of malignancies or trauma. All the procedures were completed in single-stage. The clinical outcomes associated with safety and efficacy are evaluated and discussed in detail.

## Materials and methods

### Data collection

We identified patients diagnosed with eyelid defect due to excision of malignancies or trauma, and underwent subcutaneous pedicled propeller flap for reconstructing the large eyelid defect in the Department of Burns Plastic Surgery, General Hospital of Ningxia Medical University, Yinchuan, China. The clinical data mainly include age, gender, defect size of eyelid, operation time, flap size, follow time and complications. We reviewed and analyzed these data. Informed consent for publishing photos was obtained from the patients and their privacy rights were observed. This study was approved by the Ethics Committee of the General Hospital of Ningxia Medical University, Yinchuan, China.

### Surgical procedure

**Figure 1:**
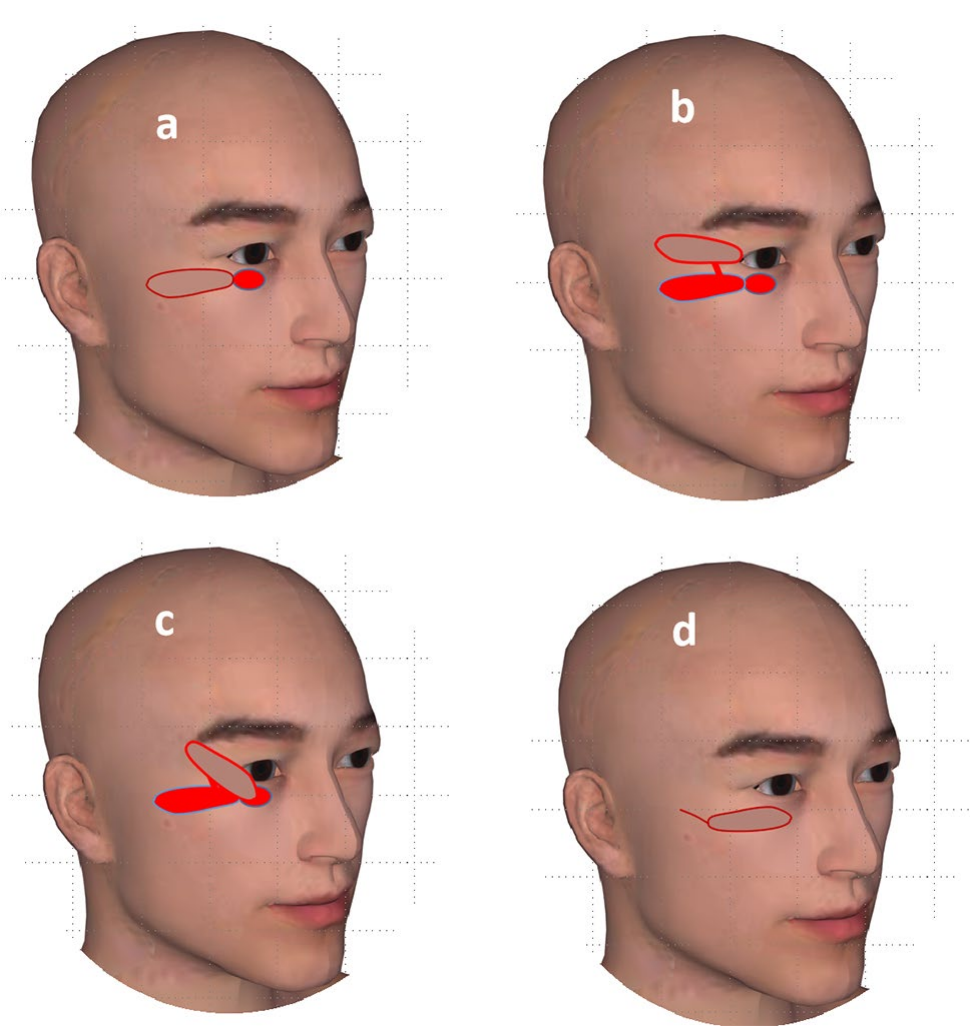
The schematic diagram of the operation. (**a**) The subcutaneous pedicled propeller flap was designed according to the eyelid defect. (**b**) The subcutaneous pedicled propeller flap was cut. (**c**) The flap was rotated for 180° to cover the defect. (**d**) The flap was sutured to the defect, and the donor site was also sutured.

The surgery was performed under general anesthesia with the patient laying in a supine position. For patient diagnosed with periocular cutaneous malignancy, we drew the excision borders according to the tumor character. In general, Mohs micrographic surgery was commonly the choice of therapy. When performing Mohs surgery, frozen examination is needed. The frozen pathology examination may be performed several times to make sure the margins were negative with minimum normal tissue excision. In our clinical work, we designed the incision for malignant tumor be more than 1.0 cm from the edge of the tumor, during which the first frozen pathology examination usually indicates a negative margin. After we performed the extended resection, we usually need to wait for about 30 min to obtain the result of the frozen pathology examination. The schematic diagram of the operation is shown in Fig. 1. Surgical resection was carefully performed. The eyelid tumor was totally resected, which left large eyelid defect. Frozen pathological results during the operation indicated that the tumor was completely removed and the margins were negative. For patients with eyelid defect due to trauma, the wound was carefully debrided, and the final defect zone was confirmed. For patients with defect of the posterior lamella of the eyelid, the composite chondromucosal graft dimension was usually used to repair the posterior lamella defect. In this study, all the patients had no defect of the posterior lamella of the eyelid.

Then we designed the subcutaneous pedicled propeller flap for reconstructing the large eyelid defect. In this study, the orbicularis oculi muscle was used as the pedicle. The orbicularis oculi muscle pedicle is a classical design which was reported by quite a few studies, and is considered to be a reliable method. Commonly, we did not use a doppler for marking as other studies reported. However, Doppler positioning may make the flap design more accurate.

The vascular network in the outer eyelid area is a branch of the superficial temporal artery, anastomosed by the zygomatic-orbital artery, the transverse facial artery, the branches of the frontal branch, and the branches of the lacrimal artery. The above-mentioned arteries form a free anastomotic arterial network in the superficial tissue of the eyelid, providing blood supply for the orbicularis oculi pedicle flap. The zygomaticorbital artery is the main branch of the superficial temporal artery in the temporal region, which is very important for the design of the temporal region skin flap, and its occurrence rate is 100%. The designed donor site was located 1 cm outside the corner of the lower eyelid on the same side as the flap rotation point. After measuring the size of the defect, we peeled away from the distal subcutaneous layer to form an ultra-thin skin flap. The anatomical level of the proximal pedicle was deep into the orbicularis oculi muscle, and part of the muscles was separated to form the orbicularis oculi muscle pedicle. The flap was made to give sufficient mobility to allow it to be easily transferred to the defect. After the subcutaneous pedicled propeller flap was cut, it was turned 180 degrees. Then the defect zone could be covered with the subcutaneous pedicled propeller flap. The flap was sutured to the defect with 6–0 PDS* II (Polydioxanone) Monofilament Synthetic Absorbable Suture. The donor site was also sutured with 6–0 PDS sutures. The wounds were covered with gauze, and we cleaned the wound every day after the surgery, which could reduce the blood scab formation, thus reducing the scar hyperplasia. After postoperative 7 days, the sutures were removed. During the patient’s discharge period, we observed patient flap blood transport and wound healing every day. After the patients were discharged, the patients were regularly followed up every month during postoperative 6 months, and every 3–6 months after postoperative 6 months.

### Ethical publication statement

We confirm that we have read the Journal’s position on issues involved in ethical publication and affirm that this study is consistent with those guidelines.

## Results

In this study, a total of 15 patients, diagnosed with large eyelid defect due to excision of malignancies or trauma, and underwent subcutaneous pedicled propeller flap for reconstructing the large eyelid defect, were included in the cases series. All the patients were aware of and agreed to the use of their data for this study. Nine patients were diagnosed with basal cell carcinoma, 3 patients with epidermoid carcinoma, and 3 patients with trauma. There 9 males and 6 females, and their average age was 33.5 ± 17.3 years old (range from 5 to 65 years). Three cases had a history of hypertension, and one patient had a history of hypertension and diabetes. None of the patients had bone invasion and metastasis of lymph nodes and distant organs. The size of tumor was ranging from 0.5–1.5 cm × 1.5–3.1 cm. The size of flap was ranging from 1.5–2.5 cm × 3.5–6.6 cm. The tumors of all the patients were completely removed, and the defects were all successfully covered with this designed flap. The average follow-up period was 9- 36 months.

There was no flap necrosis in all the cases. There were 2 patients with congestion at the distal end of the flap. After acupuncture and bloodletting, the blood supply of the flap was improved. All of the wounds were healed within 2 weeks. During the postoperative long-term follow up, no functional problems mainly including lid margin hypertrophy, corneal problems, ectropion, lower eyelid retraction because of scar contracture, or lacrimation disorder, were observed in all of the cases. There were no incidents like eyelid bulging and asymmetry of the lid margin in all cases. One mild ptosis and three slight edemas of the upper eyelid were observed in these patients. The patients’ satisfaction was evaluated using a five-point scale (excellent: 5; good: 4; fair: 3; poor: 2; bad: 1). At long-term postoperative follow up, patients’ average satisfaction score was 3.93 ± 0.73. The characteristics of patients enrolled in this study is show in Table 1.

**Table 1:** The characteristics of patients enrolled in this study.

Patient number	15	
Gender	Female	6
	Male	9
Age-year	Average	33.5 ± 17.3
	Range	5–65
Diagnosis	Basal cell carcinoma	9
	Epidermoid carcinoma	3
	Trauma	3
Basic disease	Hypertension	4
	Diabetes	1
Metastasis of tumor	0	
Tumor size (cm^2^)	Range	0.5–1.5 × 1.5–3.1
Flap size (cm^2^)	Range	1.5–2.5 × 3.5–6.6
Follow-up period-months	Range	9–36
Blood supply problems	Congestion	2
Aesthetic problems	Ptosis	1
	Edemas of eyelid	3
Satisfaction scale	Average	3.93 ± 0.73
	Range	3–5

### Representative cases

#### Case 1

**Figure 2:**
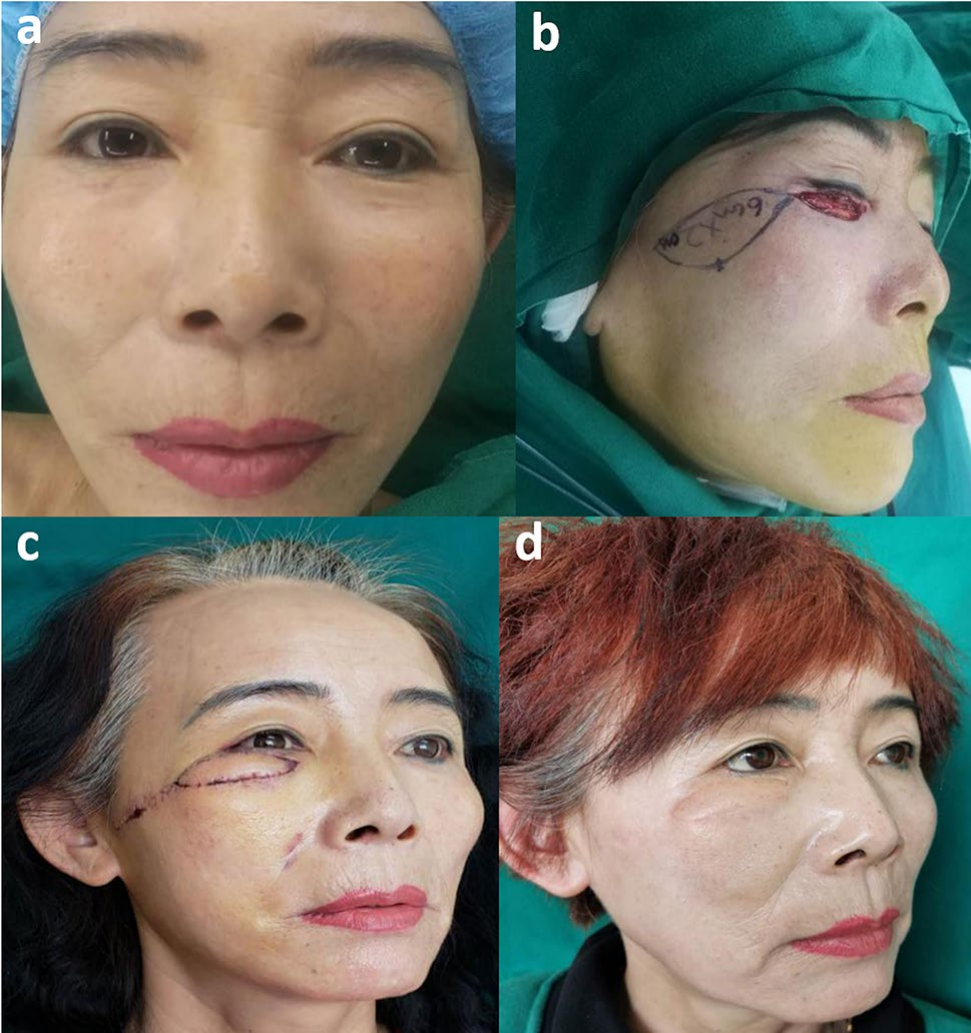
(**a**, **b**) A 52 years old female diagnosed with basal cell carcinoma. The tumor was resected and she was performed this subcutaneous pedicled propeller flap for reconstructing the large eyelid defect. (**c**) When the sutures were removed 7 days after the operation, the flaps survived well. (**d**) After 1 year of follow-up, the scar was not obvious, the appearance was satisfactory, there was no tumor recurrence, and no traction deformity.

This patient was a woman, 52 years old (Fig. 2). The lower eyelid mass of the right eye was observed for more than 2 years. A pathological biopsy was performed in an outside hospital, suggesting basal cell carcinoma. There were two 0.5 cm × 0.5 cm tumors under the lower lid of the right eye. The color was darker than normal skin, the boundary was clear, and the skin surface was slightly protruding. She was performed this subcutaneous pedicled propeller flap for reconstructing the large eyelid defect. She was given blood circulation treatment after the surgery. When the sutures were removed 7 days after the operation, the flaps survived well. After 1 year of follow-up, the scar was not obvious, the appearance was satisfactory, there was no tumor recurrence, and no traction deformity.

#### Case 2

**Figure 3:**
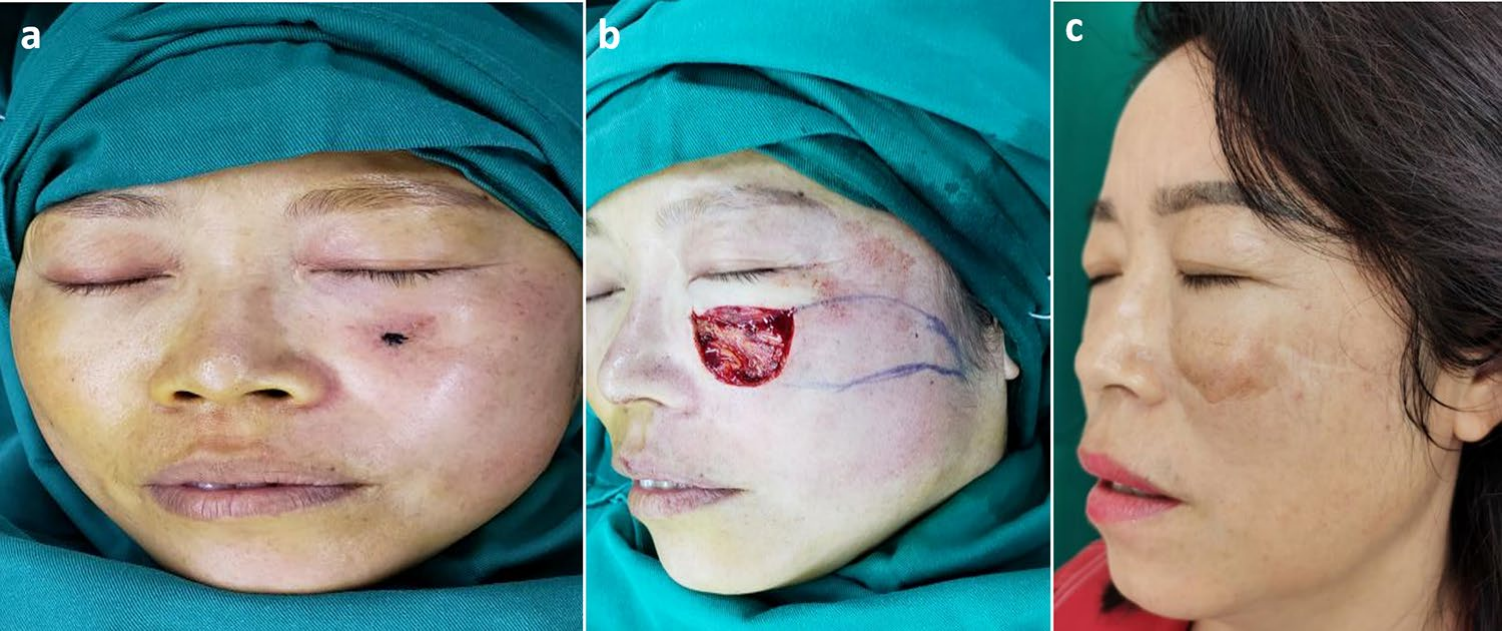
(**a**) A 48-year-old woman had a basal cell carcinoma of the left lower eyelid. (**b**) The tumor was extensively excised, and the flap was designed. (**c**) Good appearance and normal function of the eyelid was obtained at the long-term follow up.

A 48-year-old woman had a basal cell carcinoma of the left lower eyelid (Fig. 3). After the carcinoma was extensively excised, there existed a large defect of lower eyelid. The subcutaneous pedicled propeller flap was used to cover the large defect. After more than 1 year postoperative, the scar was not obvious, with satisfactory appearance, no tumor recurrence, and no pulling deformity.

#### Case 3

**Figure 4:**
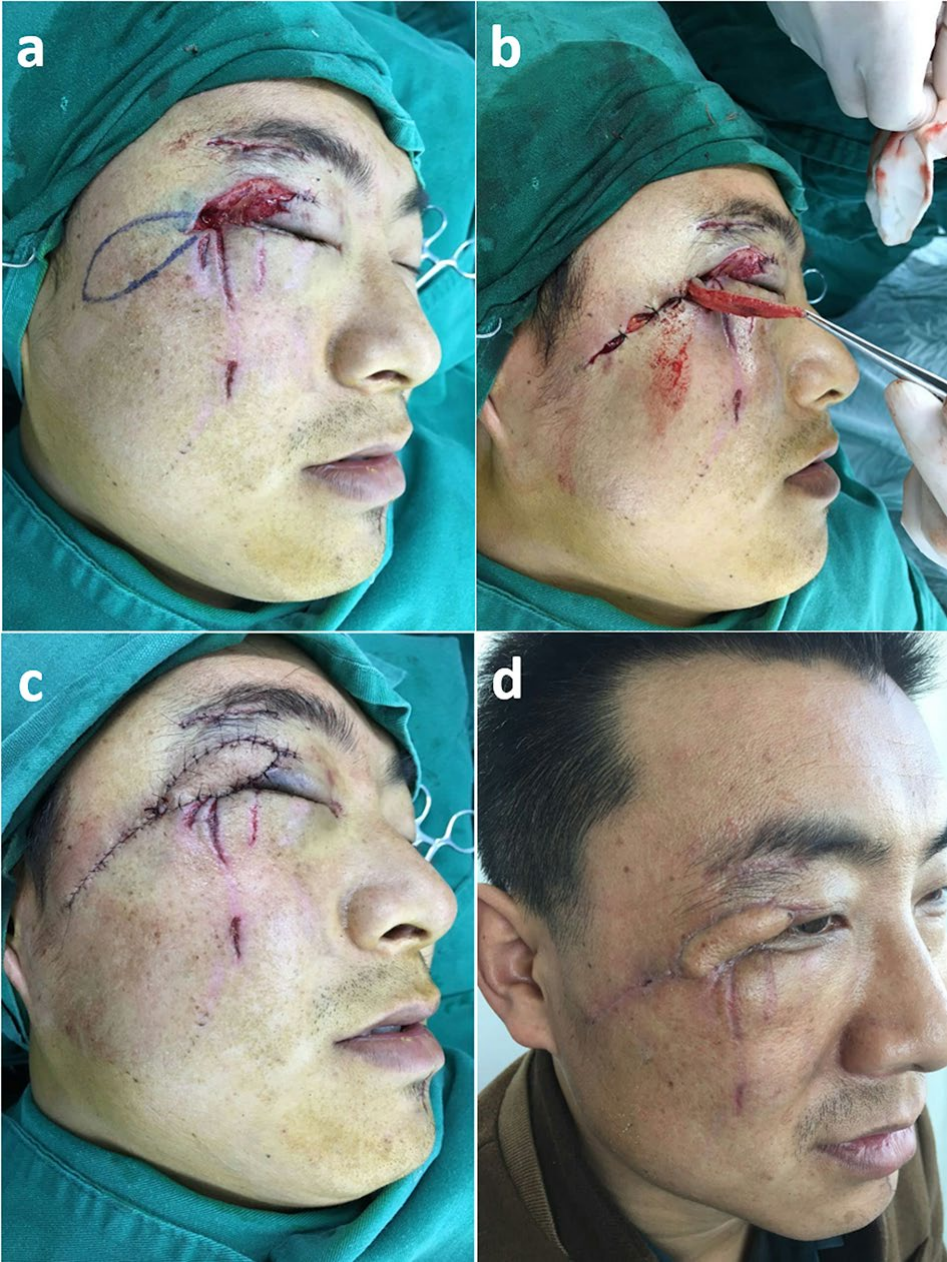
(**a**) A 49-year-old man had a large defect of upper eyelid due to car accident. (**b**, **c**) After a thorough debridement, the subcutaneous pedicled propeller flap was designed to reconstruct the large defect. (d) After postoperative 1 week, the sutures were removed and the wound healed well.

A 49-year-old man had a large defect of upper eyelid due to car accident (Fig. 4). After a thorough debridement, the subcutaneous pedicled propeller flap was designed to reconstruct the large defect. After postoperative 1 week, the sutures were removed and the wound healed well. We advised the patient to have a second surgery to make the appearance better, but the patients reported he was satisfied with the outcome and refused to undergo a second surgery.

#### Case 4

**Figure 5:**
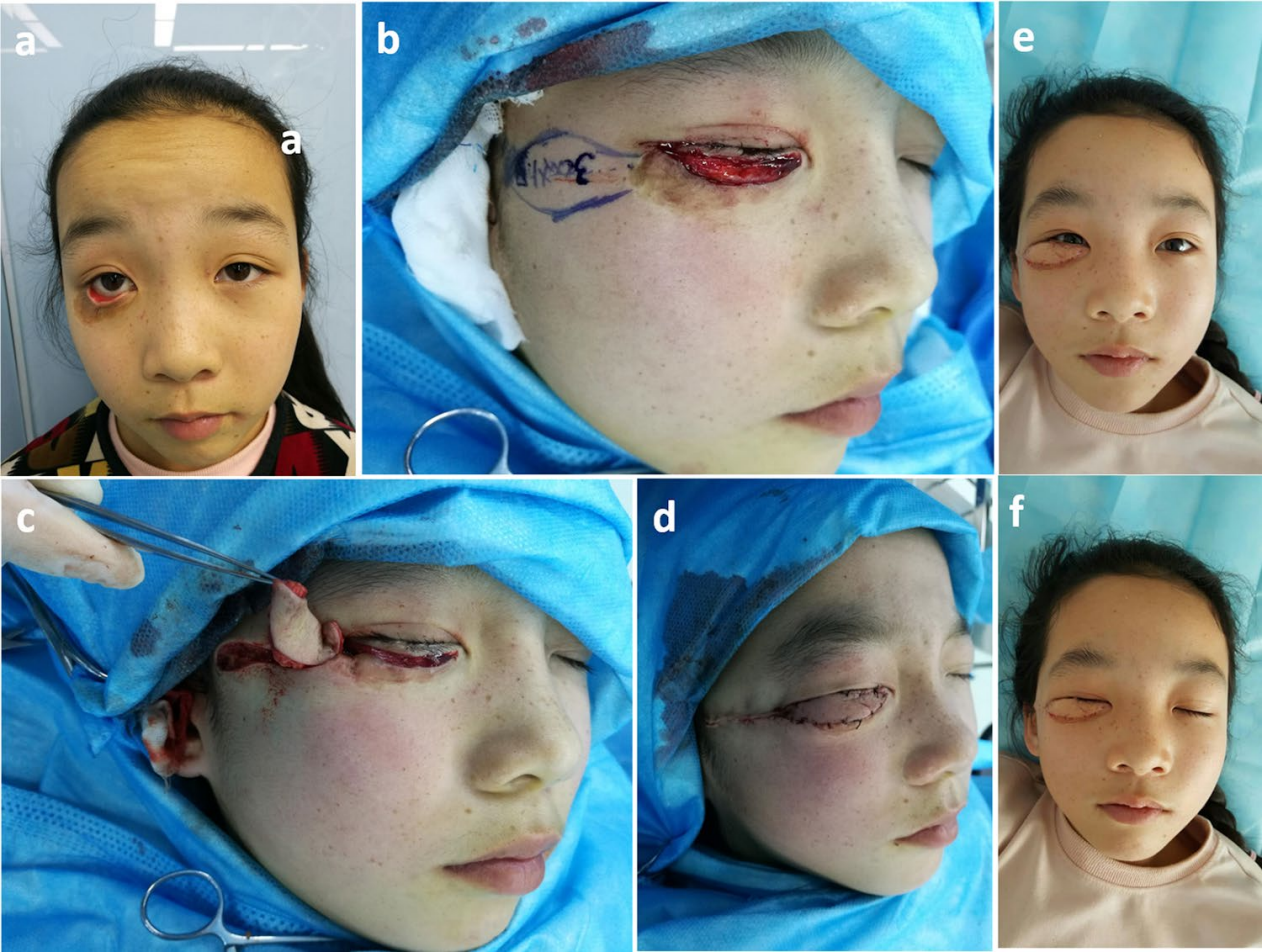
(**a**, **b**) A 5-year-old girl had a lower eyelid defect caused by a fall. After she was treated with skin grafting on the lower eyelid in the outer hospital, the skin contracture caused the lower eyelid ectropion. (**c**, **d**) We loosed the contracture of the tissue and designed the subcutaneous pedicled propeller flap to correct the defect. (**e**, **f**). The skin contracture and lower eyelid ectropion were successfully corrected and the wound healed well.

A 5-year-old girl had a lower eyelid defect caused by a fall (Fig. 5). After she was treated with skin grafting on the lower eyelid in the outer hospital, the skin contracture caused the lower eyelid ectropion. She was taken to our hospital for further treatment. We loosed the contracture of the tissue and designed the subcutaneous pedicled propeller flap to correct the defect. The skin contracture and lower eyelid ectropion were successfully corrected.

## Discussion

The eyelid has very important functions such as protecting the cornea, moisturizing the surface of the eye, removing foreign bodies, and closing and opening the eyelid^17,18^. Even some small changes of eyelid may have great impact on the patient’s facial appearance and eye functions^19^. Tumor resections, trauma and congenital defects are not rare that commonly produce large defect of eyelid^20,21^.

Recently, many techniques have been explored to reconstruct the eyelid. However, it is still a difficult problem to repair and reconstruct the large defect around the eye. Due to the large defect, traction deformity may occur after direct suture^22^. Reconstruction with free skin flaps requires microscopic anastomosis of the blood vessels, which is difficult to operate^23,24^. Vasospasm and flap necrosis are prone to occur after surgery, and the skin texture and color of the donor and recipient areas are different^12,25^. Although skin grafting alone can meet the needs of its size, it fails to repair the defective soft tissue, so it cannot restore its normal shape and function^26,27^. Local skin flaps are used for reconstruction by using adjacent or similar tissues at the defect site, and their tissue structure, color and texture are closest to the defect tissue, so it is the preferred method for defect repair after enlarged resection of malignant tumors around the eye^28–30^. In addition, the looseness of the eyelid and surrounding tissues and the good blood supply of the facial tissues also provide favorable conditions for the repair of local flaps of eyelid defects^31,32^. Commonly used types of local flaps include: advance flaps, including AT flaps, Burow wedge-shaped flaps; pivotal flaps, including modified diamond flaps, translocation flaps, and subcutaneous tissue pedicle flaps, including the orbicularis oculi muscle, pedicled lower eyelid kite flap, nasolabial flap pedicled with subcutaneous tissue; frontal nasal flap; palpebral reflex flap, etc^33–35^. The above-mentioned local flaps which can repair large defects, mainly are subcutaneous tissue pedicle flaps^36,37^. The nasolabial fold and forehead flaps need to detect and separate anatomical perforators, which is technically difficult^38^. At the same time, the flap supply area is located in the center of the face, and the scar is obvious.

The application of the orbicular oculi muscle flap on the same side avoids the above-mentioned problems^39,40^. It is pedicled with the orbicular oculi muscle and is easy to operate. The donor site is located on the outer face of the face near the front of the ear and the scar is hidden. In this study, on the basis of previous studiess, we modified it into a large ultra-thin skin flap, so that the repaired area was enlarged compared to the front, and the ultra-thin skin flap was formed at the distal end and the surrounding tissues were excessively natural and beautiful. Ultra-thin skin flap is a kind of thin skin flap modified by reducing most of the fat on the basis of traditional random flap or axial skin flap, leaving a thin layer of fat under the subdermal vascular network. The subdermal vascular network is located at the junction of the dermal reticular layer and the subcutaneous tissue. It is the origin of dermal blood vessels and plays a major role in the blood supply of the local skin^41,42^. The density of the subdermal vascular network varies throughout the body, and the density of face and neck is larger than that of limbs and the trunk^43,44^. Therefore, the length and width ratios of the designed flaps are different. The subdermal vascular network on the face is abundant, and the ultra-thin flaps have good blood supply. It can receive the three-channel blood supply from the pedicle, the wound surface of the affected area and the wound edge, and the survival rate is high. It provides an anatomical basis for the improvement of the traditional orbicular oculi muscle flap^45,46^. Our modified orbicularis oculi muscle flap is made into an ultra-thin flap at its distal end. The blood supply part is derived from the subdermal vascular network, so it can be enlarged to 30–40 mm in width and 60–70 mm in length. It is a large flap, basically close to, or equivalent to an oversized flap. Generally, the ratio of the size of the local flap pedicle to the size of the flap is the largest to 1:3, but the modified orbicularis oculi muscle flap can reach 1:4–1:5, and can be rotated 180°at will. In local flap, venous congestion can be seen more common than arterial problems in these kinds of flaps. When we encounter venous congestion at the distal end of the flap, we use frontal massage and acupuncture for bloodletting to improve the problem of venous congestion.

In previous studies, the lateral cheek rotation flap which was reported by Mustardé, and the rotation-advancement cervicofacial flaps which were derived from Mustardé flap, were considered to be the most attractive in reconstructing medium-sized suborbital skin defects^47–49^. There are still some problems with these methods, especially in patients with no skin laxity in the cervical and facial regions. These methods create a new triangular preauricular skin defect which may need skin grafting. The distal edge of the flap may develop necrosis when it is sutured under tension. Lower lid ectropion was not rare after these surgeries. In this current study, the flap can also be used in patients with no skin laxity. Besides, the subcutaneous release range is not that large, and there is no triangular preauricular skin defect which may need skin grafting. However, for defects in the orbicularis muscle in the outer canthus, this procedure is not suitable because a vascular pedicle cannot be formed.

In this study, a total of 15 patients, diagnosed with eyelid defect due to excision of malignancies or trauma, and underwent subcutaneous pedicled propeller flap for reconstructing the large eyelid defect. The defects were all successfully covered with this designed flap. There were no incidents like eyelid bulging and asymmetry of the lid margin in all cases. The patients’ average satisfaction score was 3.93 ± 0.73. The clinical outcomes demonstrated the safety and effectiveness of this designed flap for reconstructing the large eyelid defect. The designed orbicularis oculi muscle flap has several advantages. The flap is large, so it can basically be a large flap and can cover lager defect. The flap is thin, and most of the distal end is close to a full-thickness skin, which can better avoid causing blood supply problems. The flap has good appearance, similar texture, color, thickness, and satisfactory appearance after operation. Since we make full use of the excess skin on the outside of the eye and the lower eyelid margin incision for the operation of the eye bag, the incision conceals the scar and is not obvious. The donor site is small and concealed, and no new trauma is added. Therefore, the flap is a good repair method for wound repair after resection of malignant tumors around the eye, and it is worthy of clinical promotion and application.

After all, there are still several precautions in the operation of this study. Firstly, before surgery, we should check whether there is enough skin around the defect to cover. The wounds located near the inner canthus and nose are far away, so caution is required. Besides, the size of the skin flap is closely related to the laxity of the skin. For middle-aged and elderly patients, the flap can be slightly smaller than the wound surface due to the large laxity of the skin. For patients of other age groups, the flap size should be larger than 10–30% of the wound surface to avoid suture tension affects local blood supply. What’s more, when we suture the wound, the donor area of the flap should be closed first to reduce the tension of the flap in the recipient area. We should master the separation level. The proximal pedicle should not be too thin, the subcutaneous pedicle should be wide enough, and the pedicle should not have excessive tension or serious distortion. Finally, drainage strips or drainage tubes can be placed after surgery to eliminate dead space and prevent the formation of subcutaneous hematomas. Appropriate pressure bandaging should not be too high so as not to affect the blood supply of the flap.

## Conclusion

The subcutaneous pedicled propeller flap provides satisfactory outcomes for reconstructing large eyelid defect due to excision of malignancies or trauma. Therefore, it is a simple and safe technique with advantages such as keeping good appearance as well as normal eyelid functions.

## Data Availability

The data that support the findings of this study are available from the corresponding author upon reasonable request.
